# A Four-Cell-Senescence-Regulator-Gene Prognostic Index Verified by Genome-Wide CRISPR Can Depict the Tumor Microenvironment and Guide Clinical Treatment of Bladder Cancer

**DOI:** 10.3389/fimmu.2022.908068

**Published:** 2022-07-11

**Authors:** Jian-Xuan Sun, Chen-Qian Liu, Jin-Zhou Xu, Ye An, Meng-Yao Xu, Xing-Yu Zhong, Na Zeng, Si-Yang Ma, Hao-Dong He, Zong-Biao Zhang, Shao-Gang Wang, Qi-Dong Xia

**Affiliations:** Department and Institute of Urology, Tongji Hospital, Tongji Medical College, Huazhong University of Science and Technology, Wuhan, China

**Keywords:** senescence, tumor microenvironment, tumor mutation burden, bladder cancer, immunotherapy, chemotherapy

## Abstract

Bladder cancer (BCa) is the 10th most commonly diagnosed cancer worldwide, and cellular senescence is defined as a state of permanent cell cycle arrest and considered to play important roles in the development and progression of tumor. However, the comprehensive effect of senescence in BCa has not ever been systematically evaluated. Using the genome-wide CRISPR screening data acquired from DepMap (Cancer Dependency Map), senescence genes from the CellAge database, and gene expression data from The Cancer Genome Atlas (TCGA), we screened out 12 senescence genes which might play critical roles in BCa. A four-cell-senescence-regulator-gene prognostic index was constructed using the least absolute shrinkage and selection operator (LASSO) and multivariate COX regression model. The transcriptomic data and clinical information of BCa patients were downloaded from TCGA and Gene Expression Omnibus (GEO). We randomly divided the patients in TCGA cohort into training and testing cohorts and calculated the risk score according to the expression of the four senescence genes. The validity of this risk score was validated in the testing cohort (TCGA) and validation cohort (GSE13507). The Kaplan–Meier curves revealed a significant difference in the survival outcome between the high- and low-risk score groups. A nomogram including the risk score and other clinical factors (age, gender, stage, and grade) was established with better predictive capacity of OS in 1, 3, and 5 years. Besides, we found that patients in the high-risk group had higher tumor mutation burden (TMB); lower immune, stroma, and ESTIMATE scores; higher tumor purity; aberrant immune functions; and lower expression of immune checkpoints. We also performed gene set variation analysis (GSVA) and gene set enrichment analysis (GSEA) to investigate the interaction between risk score and hallmark pathways and found that a high risk score was connected with activation of senescence-related pathways. Furthermore, we found that a high risk score was related to better response to immunotherapy and chemotherapy. In conclusion, we identified a four-cell-senescence-regulator-gene prognostic index in BCa and investigated its relationship with TMB, the immune landscape of tumor microenvironment (TME), and response to immunotherapy and chemotherapy, and we also established a nomogram to predict the prognosis of patients with BCa.

## Introduction

Bladder cancer (BCa) is the 7th most commonly diagnosed cancer in men and has the 10th highest incidence in the whole population around the world, with approximately 550,000 new cases annually worldwide (1) and 85,694 in China in 2020 (2). It has the 13th highest yearly mortality among all cancers and caused more than 165,000 in 2012, and the number is still rising despite tremendous treatment efforts (3). Tobacco smoking and occupational exposure to carcinogens are the major risk factors for BCa, while genetic factors seem to have little impact (3, 4). Urothelial carcinoma accounts for more than 90% of all cases, and BCa could be divided into non-muscle-invasive bladder cancer (NMIBC) and muscle-invasive bladder cancer (MIBC) according to whether the tumor tissue invades the muscle layer of the bladder. Approximately 75% of BCa is confined to the mucosa (stage Ta, CIS) or submucosa (stage T1) and defined as NMIBC; the remaining 25% is attributed to MIBC (4, 5). The primary treatment for NMIBC is transurethral resection of the bladder (TURB) accompanied by intravesical chemotherapy or intravesical bacillus Calmette-Guérin (BCG) immunotherapy after TURB to prevent recurrence and progression (4). As for MIBC, radical cystectomy (RC) and pelvic lymph node dissection (PLND) are the main treatments, and cisplatin-based chemotherapy has become the standard procedure for disease management (6). The traditional methotrexate, vinblastine, doxorubicin, and cisplatin (MVAC) chemotherapy plan has gradually been substituted by gemcitabine plus cisplatin (GC) for its similar efficacy and improved tolerability. Immunotherapy using immune checkpoint inhibitors (ICBs) and pre- and postoperative radiotherapy are also possible alternatives for platinum-ineligible patients (5–7). Other new small-molecule drugs such as OICR-9429 have also been proved to be effective in the treatment of bladder cancer, which can enhance the antitumor effect of cisplatin or immunotherapy in BCa (8). Discovering new therapeutic strategies, choosing appropriate candidates for immunotherapy using ICDs, and predicting the therapeutic efficacy for immunotherapy and chemotherapy are the major concerns in bladder cancer treatment.

Discovered by Hayflick and Moorhead in 1961 (9), cellular senescence is defined as a state of permanent cell cycle arrest induced by various *in vitro* or *in vivo* stimuli and characterized by aberrant cellular structure and morphology, as well as the activation of several hallmark signaling pathways such as DNA damage response (DDR), apoptosis resistance, cyclin-dependent kinase (CDK) inhibition, endoplasmic reticulum (ER) stress, and increased secretion of proinflammatory and tissue-remodeling factors (10, 11). According to the different stimuli, cellular senescence can be divided into several subtypes including replicative senescence, DNA damage-induced senescence, oncogene-induced senescence (OIS), and chemotherapy-induced senescence. Cellular senescence is a double-edged sword for human health. On the one hand, senescence can promote tissue remodeling and repair and function as a powerful safeguard to prevent abnormal proliferation and tumorigenesis. On the other hand, as an important part of aging, the aberrant and excessive accumulation of senescent cells is also connected with age-related disorders like degenerative diseases and cancer (11, 12).

Cellular senescence plays a dual role in the initiation, growth, and progression of tumor. The activation of oncogenes or loss of tumor suppressors will induce OIS and tumor-suppressor gene (TSG) loss–induced senescence, respectively, which can arrest the cell cycle and prevent tumorigenesis (13). Senescent tumor cells can also modulate the tumor microenvironment (TME) through the non-cell autonomous regulation of senescence called senescence-associated secretory phenotype (SASP). Through SASP, senescent cells can induce paracrine senescence and transform surrounding non-senescent cells into senescent cells by secreting pro-inflammatory cytokines, chemokines, growth factors, and proteases like IL-6, IL-8, and TGF-β, recruiting and activating immune cells in TME, which can result in both antitumor- and tumor-promoting effects. On the one hand, the innate and adaptive immune cells like M1 macrophages and natural killer (NK) cells can clear the tumor cells and promote their senescence by secreting IFN-γ and TNF-α, thus limiting tumor growth. On the other hand, senescent tumor cells can also attract and activate myeloid-derived suppressor cells (MDSCs) and M2 macrophages *via* SASP, thus affecting the clearance of senescent tumor cells and promoting tumor progression and vascularization, which is called maladaptive senescence (11, 13, 14). Since cellular senescence has a role in limiting tumor growth and development, it has been considered as a potential therapeutic target for tumor treatment. Multiple commonly used chemotherapy drugs such as bleomycin or doxorubicin can induce senescence to exert an antitumor effect. Moreover, several other pro-senescence therapy strategies such as telomerase inhibition, immunotherapy, and SASP reprogramming have also been put forward (13). Therefore, it is important for us to figure out the comprehensive role of senescence in tumorigenesis and TME shaping to benefit as much as possible from interventions without incurring toxicities.

In recent years, several studies have focused on the role of senescence in BCa. Xia et al. have found that berberine can induce BCa cell senescence *via* inhibiting Janus kinase 1 (JAK1)-STAT3 signaling and upregulating miR-17-5p (15). Chen et al. found a novel cellular senescence gene called SENEX, which could promote regulatory T cell (Treg) accumulation in aged urinary BCa (16). Moreover, Wang and colleagues also found that regulator of cullins-1 (ROC1) could promote NMIBC progression through anti-senescence and was linked with poor prognosis (17). However, the previous studies were limited, and all focused on few senescence genes. The role of senescence in BCa has never been systematically evaluated, and the relationship between senescence and the prognosis of BCa remains obscure.

Therefore, here, using the genome-wide CRISPR screening data acquired from DepMap (Cancer Dependency Map), senescence genes from the CellAge database, and gene expression data from The Cancer Genome Atlas (TCGA), we screened out 12 senescence genes which might play key roles in BCa. Then, we successfully constructed a four-cell-senescence-regulator-gene prognostic index using the least absolute shrinkage and selection operator (LASSO) and multivariate COX regression model and investigated its relationship with tumor mutation burden (TMB) and TME immune cell infiltration. Its correlations with the prognosis of BCa and the efficacy of immunotherapy and chemotherapy were also investigated. We also established a nomogram using the senescence risk score and other clinical characteristics to predict the prognosis of patients with BCa.

## Methods

### Obtaining BLCA Patient Bulk-Seq Data and Identifying Essential Cell Senescence Regulator Genes in BLCA

The RNA bulk-seq data and corresponding clinical information were retrieved and downloaded from the GDC_Data Portal (https://portal.gdc.cancer.gov/), and the external validation set GSE13507 was obtained from the Gene Expression Omnibus (https://www.ncbi.nlm.nih.gov/geo/). More importantly, the genome-wide CRISPR screening results of bladder cancer cell lines were acquired from the DepMap Database (https://depmap.org/portal/download/), and the importance for each candidate gene was weighted by the algorithm of CERES score. Notably, the genes with CERES score <-1 across of 75% of BLCA cell lines were screened as candidate genes (18, 19). Following this, the differentially expressed genes (DEG) analysis of these candidate genes was carried out between paired tumor sample and normal sample in TCGA_BLCA cohort. FDR <0.05 and log_2_FC >0 were set as the filter threshold to identify the essential BLCA genes. Also, 279 cell senescence regulator genes were obtained from the CellAge database (https://genomics.senescence.info/cells/) (20). Finally, we took an intersection of the essential BLCA genes and cell senescence regulator genes and got a total of 12 essential cell senescence regulator genes (ECSRGs) in BLCA for further analysis.

### Comprehensive Analysis of the Essential Cell Senescence Regulator Genes in BLCA

Having got 12 ECSRGs in BLCA, we firstly conducted a comprehensive analysis to investigate the role of these ECSRGs. Both the expression profile and copy number variation (CNV) profiles were used for further analysis. Then, the differentially expressed analysis and expression correlation analysis of these 12 ECSRGs between tumor tissues and normal tissues (or normal adjuvant tumor tissues) were carried out to check the expression patterns of these ECSRGs in TCGA_BLCA cohort and GSE13507 cohort. Also, the copy number variation of these ECSRGs was further investigated. Finally, the 12 ECSRGs were uploaded to the STRING database (https://string-db.org/cgi/input.pl) to explore the protein–protein interaction network, and Cytoscape (version 3.8.2) was used to visualize the PPI network of these 12 ECSRGs.

### Elimination of the Batch Effects, Sample Random Grouping, Construction, and Verification of the Prognostic Cell Senescence Index

We merged the expression matrix of TCGA_BLCA cohort and GSE13507 cohort, and the batch effects were eliminated by the combat algorithm using the “sva” package in R program (21). Notably, only 11 ECSRGs were covered in both TCGA_BLCA cohort and GSE13507 cohort, and they were CDK1, PSMD14, CHEK1, PSMB5, MAD2L1, RUVBL2, GAPDH, PRPF19, TPR, RAD21, and SUPT5H. Then we randomly split the samples with a ratio of 1:1 in TCGA_BLCA cohort into train group and validation group. The least absolute shrinkage and selection operator (LASSO) method was conducted in the training group to screen the appropriate variables of the above 11 ECSRGs. Following this, the multivariate cox regression of the left variables was performed in the training group to establish a prognostic cell senescence index (PCSI). Meanwhile, each sample in TCGA_validation cohort and GSE13507 cohort acquired a PCSI according to the following formula:


$$PCSI=\sum_{i=1}^{n}coef\left(i\right)\cdot expr\left(i\right).$$

Among them, coef(i) is the coefficient of the ith ECSRGs in PCSI, and expr(i) is the normalized expression values of the ith ECSRGs. The medium value of the PCSI in TCGA_training cohort was set as the threshold, the higher PCSI was defined as high risk, and the lower PCSI was low risk; each patient in both TCGA cohort and GSE13507 cohort obtained a risk stratification level according to their PCSI.

The log-rank test-based survival analysis and Kaplan–Meier method-based survival curves were used to investigate the survival differences between high-PCSI patients and low-PCSI patients in TCGA_training cohort, TCGA_test cohort, and GSE13507 validation cohort. Both receiver operating characteristic curve (ROC) and univariate-cox regression were carried out to further explore the prognostic capability of the PCSI. Following this, considering that these three cohorts were independent between each other, we performed heterogeneity test and meta-analysis of the hazard ratio (HR) in these three cohorts to objectively check the summary prognostic role of the PCSI. In addition, we would like to improve the prognostic accuracy of the PCSI. Thus, we combined the PCSI with other commonly used clinicopathological characteristics including age, gender, grade, and stage to assemble a nomogram; the 1-, 3-, and 5-year calibration curves and 5-year ROC curves were plotted to examine the efficacy of the nomogram.

### Mutation Atlas, Immune Infiltrations, Immune Checkpoints, and Immune-Related Functions Between High- and Low-PCSI Patients

Having verified the efficacy of the PCSI and PCSI-based nomogram, we were interested in the potential mechanisms behind the differential PCSI groups. Considering that the expression matrix of TCGA was more complete, the further function analysis was all based on TCGA cohort. The mutation profiles of TCGA_BLCA cohort were retrieved and downloaded from GDC_Data_Portal (https://portal.gdc.cancer.gov/), then the TMB of each patient is calculated and the mutation profiles were sorted according to the PCSI levels. A differential mutation atlas between high-/low-PCSI groups was visualized as waterfall plots, and the χ^2^ test was carried out to check the significantly differentially mutated genes between high-/low-PCSI groups. Following this, we combined the PCSI with TMB to stratify the patients and check the prognostic effects of these two indicators.

Considering that there existed a total of seven algorithms to quantify the immune infiltration according to the expression matrix, we downloaded the immune infiltration calculated by TIMER, CIBERSORT, CIBERSORT-ABS, QUANTISEQ, MCPCOUNTER, XCELL, and EPIC methods from the TIMER 2.0 database (http://timer.cistrome.org/) (22). Then both correlation tests and differential immune infiltration analysis were performed to investigate the immune infiltration between differential PCSI groups. Besides, the immune-related scores and immune-related functions were also investigated by the ESTIMATE algorithm (23) and single-sample gene set enrichment analysis (ssGSEA) algorithm (24, 25). The immune checkpoint expression matrix was extracted and used for further DEG analysis and correlation tests.

### GSEA, GSVA, Drug Response, External Validation in Immunotherapy Cohort IMvigor-210, and the Histological Verification in Protein Levels

Gene set enrichment analysis (GSEA) was conducted by the “clusterProfiler” package in R program (26, 27), and HALLMARK gene sets were set as the enrichment gene sets. Also, gene set variation analysis (GSVA) was conducted by the “GSVA” package in R program (28). The HALLMARK gene sets, KEGG_signaling pathways, programmed cell death-related gene sets, and cell senescence-related gene sets were separately used for GSVA, and the correlation between the PCSI levels and the activated levels of these gene functions or pathways was examined by the Spearman test. Notably, all the gene sets were searched and downloaded from MSigDb (https://www.gsea-msigdb.org/gsea/msigdb/).

The drug response to chemotherapy or targeted therapy was predicted by the ProPhetic algorithm, and then the drug sensitivity to chemotherapy or targeted therapy (summarized by the IC50) was compared between high-PCSI and low-PCSI patients. Moreover, the drug response to immunotherapy was summarized by the Tumor Immune Dysfunction and Exclusion (TIDE) scores in the TIDE database (http://tide.dfci.harvard.edu/) (29), then the differential immune escape potentials (TIDE scores) between high-/low-PCSI groups were investigated by the Wilcoxon test. More importantly, we added an external bladder cancer anti-PD-L1 immunotherapy cohort IMvigor 210 (30, 31). Each patient’s PCSI in IMvigor210 was calculated by the same method, and the differential PCSI distribution between differential response to the anti-PDL1 immunotherapy groups (CR/PR or PD/SD) was compared by the Wilcoxon test. Finally, we further searched the Human Protein Atlas (https://www.proteinatlas.org/) (32) for the histological verification in protein levels of the ECSRGs enrolled in the PCSI model between bladder tumor tissues and normal bladder tissues.

## Results

### Identification of 12 Vital Differentially Expressed Senescence Genes in Bladder Cancer

**Figure 1:**
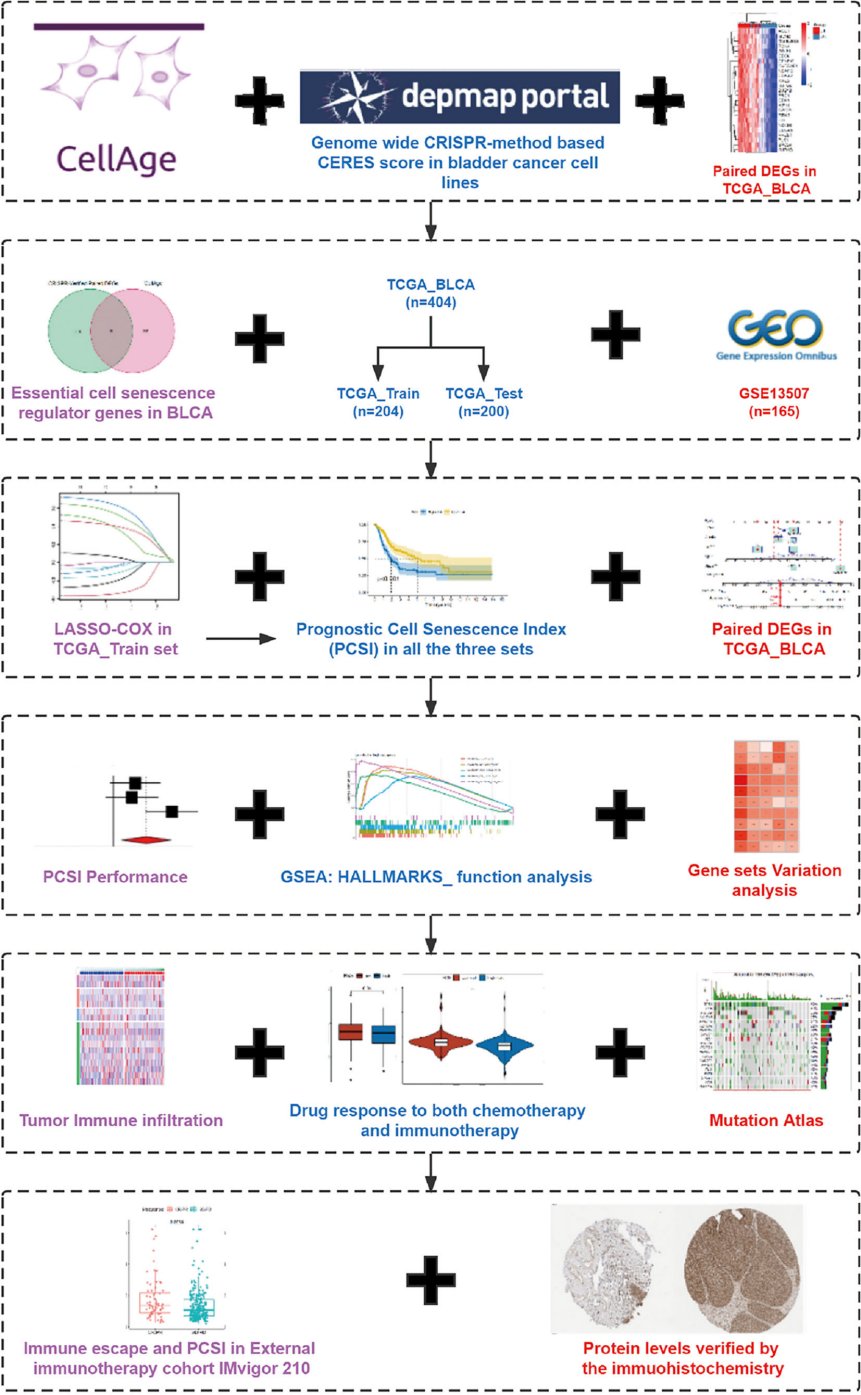
The flowchart and graphic abstract of this study.

**Figure 2:**
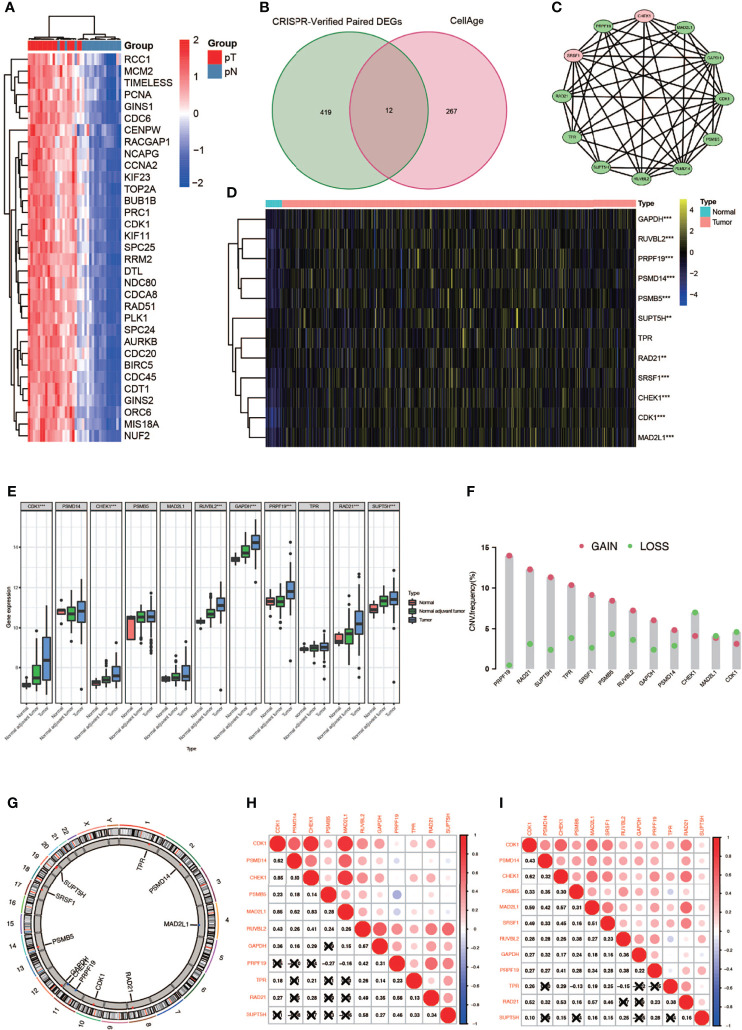
Identification of 12 vital differentially expressed senescence genes in bladder cancer. **(A)** Genome-wide CRISPR verified paired differentially expressed gene (DEGS) in TCGA database. **(B)** Venn diagram of DEGs and senescence genes in the CellAge database. **(C)** Relationship among the 12 genes. **(D)** Twelve genes were differentially expressed in normal and tumor tissues (* P < 0.05; ** P < 0.01; *** P < 0.001). **(E)** Differentially expressed 12 genes in normal, normal adjuvant tumor, and tumor tissues (** P < 0.01; *** P < 0.001). **(F)** CVN diagram of 12 genes. **(G)** The location of these 12 genes on the chromosome. **(H, I)** Correlation between 12 genes in TCGA (left) and GEO (right) databases.

A complete flowchart of the analysis process is shown in **Figure 1**. From the beginning, we extracted the genes, which was vital to the viability of bladder cancer cell lines verified by genome-wide CRISPR knockout in the DepMap database and screened out differentially expressed genes (DEGs) in normal tissues and bladder cancer tissues using the gene expression data from TCGA database (**Figure 2A**; **Supplementary Table 1**). Then we obtained the senescence genes in the CellAge database (**Supplementary Table 2**) and intersected these genes with the DEGs, and 12 essential cell senescence regulator genes (ECSRGs) were finally sorted out (**Figure 2B**). Among the 12 ECSRGs, two (SRSF1 and CHEK1) were senescence promotors and the others were senescence inhibitors. Then we analyzed the interaction among these 12 ECSRGs and found that they were tightly interacted with each other (**Figure 2C**). Later we found that the expression of these 12 ECSRGs increased gradually in normal tissues, normal adjacent tumor tissues, and tumor tissues (**Figures 2D, E**). The location of these 12 ECSRGs on the chromosome is shown in **Figure 2G**. Moreover, we also performed copy number variation (CVN) analysis of these genes and found that most alterations were loss in copy number (**Figure 2F**). Besides, the correlation among the 12 genes was illustrated after analyzing data retrieved from TCGA (**Figure 2H**) and GEO (**Figure 2I**) databases. Above all, we successfully found 12 vital ECSRGs for further analysis.

### Construction and Verification of the Prognostic Cell Senescence Index

**Figure 3:**
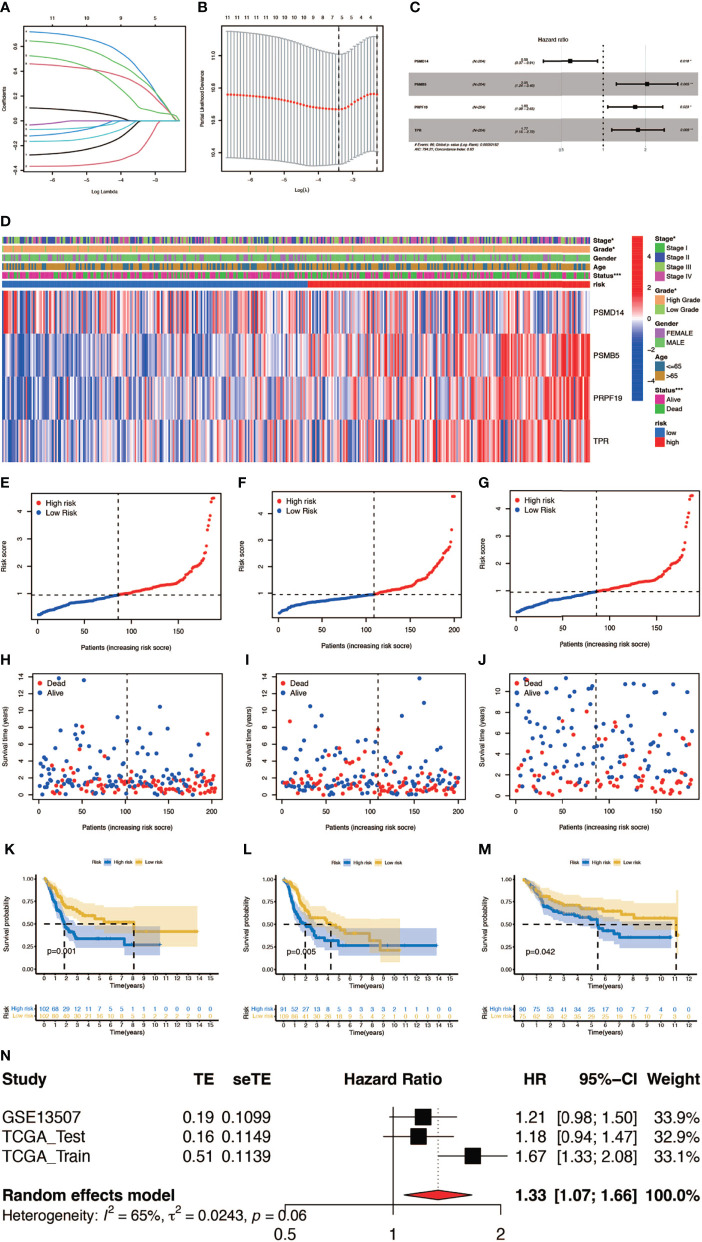
Construction and verification of the prognostic index. **(A, B)** Result of LASSO regression analysis. **(C)** Hazard ratio of each gene after multivariate Cox regression analysis. **(D)** The distribution of clinical characteristics of patients in the high- and low-risk score groups. **(E–G)** Risk scores of each patient in the training cohort, testing cohort, and verification cohort. **(H–J)** Survival status of each patient in the training cohort, testing cohort, and verification cohort. **(K–M)** Kaplan–Meier survival analyses of each patient in the training cohort (P = 0.001, log-rank test), testing cohort (P = 0.005, log-rank test), and verification cohort (P = 0.042, log-rank test). (n) Meta-analysis of the training cohort, testing cohort, and verification cohort (*P < 0.05; **P < 0.01; ***P < 0.001).

**Figure 4:**
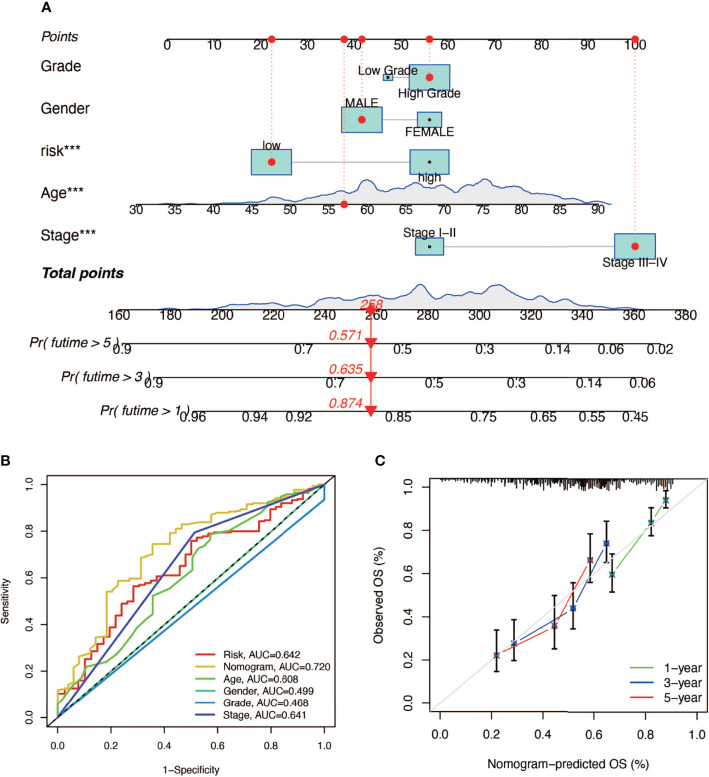
The establishment of a nomogram predicting the prognosis of patients with BCa. **(A)** Nomogram of a randomly selected patient (*** P < 0.001). **(B)** AUC curve of the nomogram. **(C)** Calibration plot of 1-, 3-, and 5-year overall survival.

In order to construct a prognostic index, we obtained 404 samples of BCa from TCGA database and divided them into two groups as training cohort (N = 204) and testing cohort (N = 200). The basic characteristics of the patients included are shown in **Table 1**. Then we used the LASSO regression and screened out five genes for further analysis (**Figures 3A, B**) and then sifted four genes from them through multivariate Cox regression using the gene expression data in the training cohort (**Figure 3C**). These four genes were PSMD14 (HR = 0.58, 95% CI = 0.37–0.91, P = 0.018), PSMB5 (HR = 2.05, 95% CI = 1.24–3.40, P = 0.005), PRPF19 (HR = 1.69, 95% CI = 1.08–2.65, P = 0.023), and TPR (HR = 1.77, 95% CI = 1.15–2.72, P = 0.009), and their interactions are shown in **Supplementary Figure 1**. The detailed information of these four genes is exhibited in **Table 2**. To evaluate the effectiveness of this prognostic index, we also obtained 165 samples from the GEO database (GSE13507) as validation cohort. We calculated the risk score according to the expression of the four senescence genes and divided the patients in these three cohorts into high- and low-risk score groups according to the median risk score as the cut-off value acquired from the training cohort (**Figures 3E–G**). The distribution of clinical characteristics of patients in the high- and low-risk score groups is shown in **Figure 3D**. Moreover, we also evaluated the survival status of each patient in three cohorts (**Figures 3H–J**). All these results revealed that patients in the high-risk score group seemed to have the worse clinical outcome than those in the low-risk group. Subsequently, we conducted Kaplan–Meier survival analyses and plotted survival curves (**Figures 3K–M**), which indicated that patients in the high-risk score group had worse survival outcome than those in the low-risk score group. The area under the curve (AUC) was used to check the effectiveness of the prognostic index (**Supplementary Figure 2**). Besides, the result of meta-analysis based on the three cohorts illustrated that the risk score was in good validity (HR = 1.33, 95% CI = 1.07–1.66, P = 0.06) (**Figure 3N**). At last, we established a nomogram using the senescence risk score and other clinical characteristics (age, gender, stage, and grade) to predict the survival probability of patients with BCa in 1, 3, and 5 years, respectively. Moreover, we randomly selected a patient and calculated that his survival probability in 5, 3, and 1 year were 57.1%, 63.5%, and 87.4%, respectively (**Figure 4A**). The AUC of this nomogram was 0.720 and the largest among all the models, indicating that the nomogram had better accuracy in predicting the overall survival (OS) compared with other independent factors (**Figure 4B**). Calibration plots of 1-, 3-, and 5-year OS also exhibited a good predicted accuracy (**Figure 4C**). Above, all these results suggested that the four-gene prognostic index could accurately and stably predict the survival outcome of BCa patients.

**Table 1:** The basic characteristics of included patients.

	Overall	GSE13507	TCGA_Test	TCGA_Train	P
n	569	165	200	204	
Age [mean (SD)]	67.25 (11.10)	65.18 (11.97)	69.10 (9.91)	67.12 (11.21)	0.003
Gender = female/male (%)	135/434 (23.7/76.3)	30/135 (18.2/81.8)	47/153 (23.5/76.5)	58/146 (28.4/71.6)	0.071
Grade (%)					<0.001
High grade	440 (77.3)	60 (36.4)	185 (92.5)	195 (95.6)	
Low grade	126 (22.1)	105 (63.6)	13 (6.5)	8 (3.9)	
Unknown	3 (0.5)	0 (0.0)	2 (1.0)	1 (0.5)	
T (%)					<0.001
T1	83 (14.6)	80 (48.5)	2 (1.0)	1 (0.5)	
T2	150 (26.4)	31 (18.8)	63 (31.5)	56 (27.5)	
T3	210 (36.9)	19 (11.5)	87 (43.5)	104 (51.0)	
T4	69 (12.1)	11 (6.7)	33 (16.5)	25 (12.3)	
Ta	24 (4.2)	24 (14.5)	0 (0.0)	0 (0.0)	
Unknown	33 (5.8)	0 (0.0)	15 (7.5)	18 (8.8)	
M (%)					<0.001
M0	352 (61.9)	158 (95.8)	95 (47.5)	99 (48.5)	
M1	18 (3.2)	7 (4.2)	6 (3.0)	5 (2.5)	
MX	197 (34.6)	0 (0.0)	97 (48.5)	100 (49.0)	
Unknown	2 (0.4)	0 (0.0)	2 (1.0)	0 (0.0)	
N (%)					<0.001
N0	386 (67.8)	151 (91.5)	115 (57.5)	120 (58.8)	
N1	54 (9.5)	8 (4.8)	22 (11.0)	24 (11.8)	
N2	79 (13.9)	4 (2.4)	37 (18.5)	38 (18.6)	
N3	8 (1.4)	1 (0.6)	4 (2.0)	3 (1.5)	
NX	37 (6.5)	1 (0.6)	19 (9.5)	17 (8.3)	
Unknown	5 (0.9)	0 (0.0)	3 (1.5)	2 (1.0)	
Status = dead (%)	245 (43.1)	69 (41.8)	90 (45.0)	86 (42.2)	0.787
PCSI (median [IQR])	0.95 [0.72, 1.33]	1.00 [0.69, 1.34]	0.92 [0.70, 1.26]	0.96 [0.75, 1.33]	0.658
Risk = high/low (%)	283/286 (49.7/50.3)	90/75 (54.5/45.5)	91/109 (45.5/54.5)	102/102 (50.0/50.0)	0.227

Prognostic cell senescence index (PCSI).

**Table 2:** The detailed information and corresponding coefficient of the four cell senescence regulator genes.

Symbol	Names	Coef
PSMD14	Proteasome 26S subunit, non-ATPase 14	-0.542818539222188
PSMB5	Proteasome 20S subunit beta 5	0.720137573972114
PRPF19	Pre-MRNA processing factor 19	0.5248045764904
TPR	Translocated promoter region, nuclear basket protein	0.5699966968373

### Exploring the Relationship Between Tumor Mutation Burden and Risk Score

**Figure 5:**
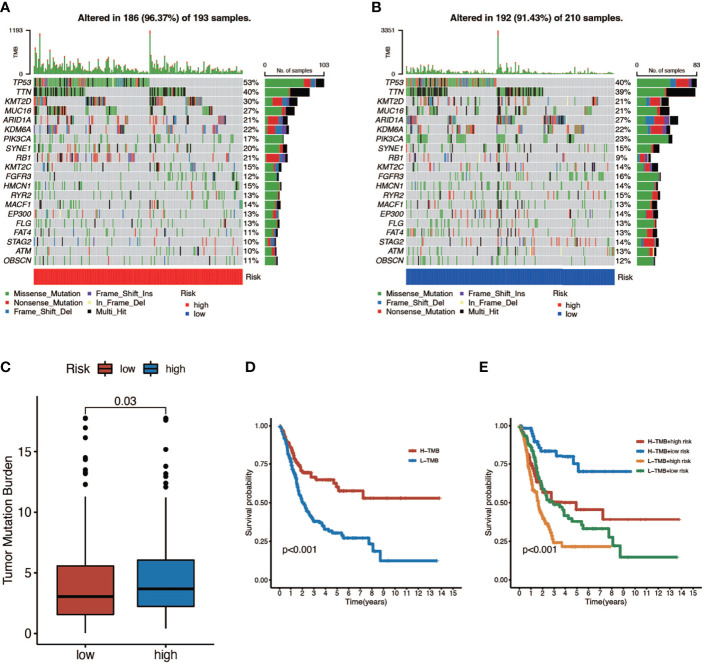
The relationship between tumor mutation burden (TMB) and risk score. **(A, B)** Mutation spectrum of high-risk (left) and low-risk (right) patients. **(C)** Comparison of TMB between patients in the high- and low-risk score groups. **(D)** Survival analyses for low- and high-TMB patient groups in TCGA-BLCA cohort using Kaplan–Meier curves (P < 0.001, log-rank test). **(E)** Survival analyses for four groups grouped according to TMB and risk score in TCGA-BLCA cohort using Kaplan–Meier curves. The high-TMB and low-risk score groups showed significantly better overall survival than the other three groups (P < 0.001, log-rank test).

Here we mapped the mutation spectrum of patients with high risk score (**Figure 5A**) and low risk score (**Figure 5B**) in TCGA and obtained six significantly differentially mutant genes including RB1, DCC, TP53, LAMA3, VPS13D, and APOB by the chi-squared test (**Supplementary Table 3**). Subsequently, we compared the TMB in high- and low-risk score patients, indicating that the TMB of patients in the high-risk score group was higher (**Figure 5C**). Survival curves also suggested that the clinical prognosis of patients with high TMB was better (**Figure 5D**), while patients with a low risk score and high TMB had the best prognosis (**Figure 5E**).

### Correlation Between Prognostic Index and Tumor Microenvironment in Bladder Cancer

**Figure 6:**
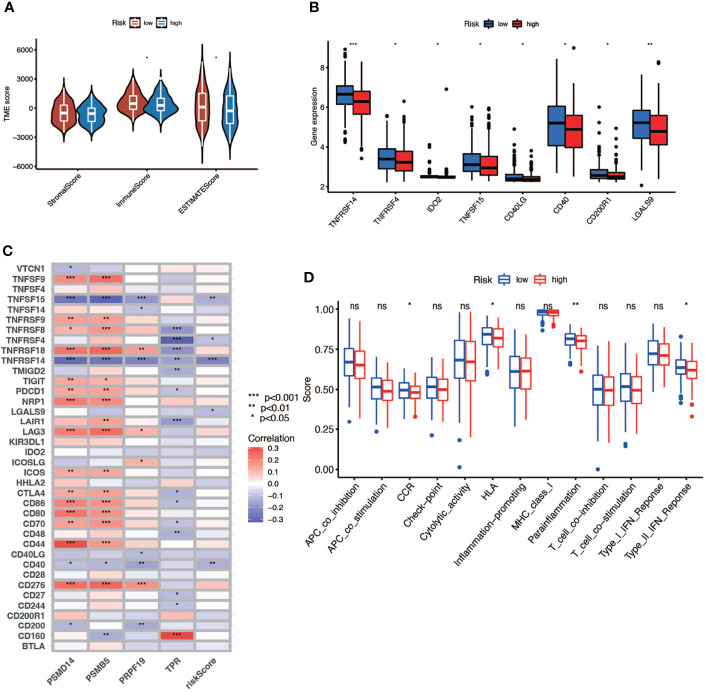
The relationship between risk score and TME. **(A)** TME scores of high- and low-risk patients (* P < 0.05; ** P < 0.01; *** P < 0.001). **(B)** Immune checkpoints expression in high- and low-risk-score patients (* P < 0.05; ** P < 0.01; *** P < 0.001). **(C)** Correlation between immune checkpoints and four genes (* P < 0.05; ** P < 0.01; *** P < 0.001). **(D)** Comparison of immune-related function of high- and low-risk-score patients (ns, no significance; * P < 0.05; ** P < 0.01; *** P < 0.001).

**Figure 7:**
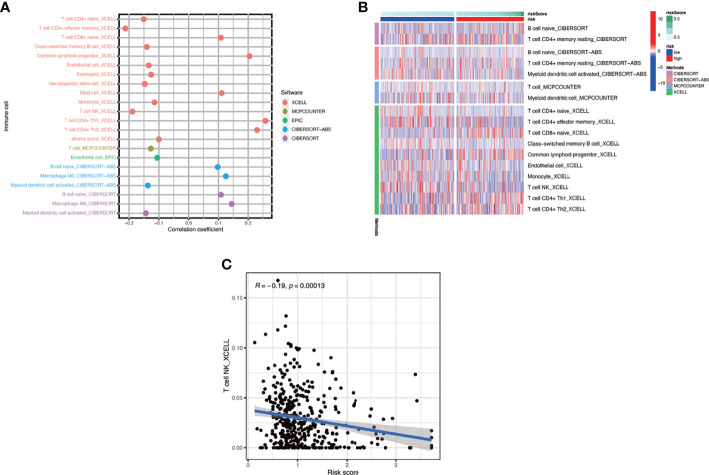
The interaction of risk score and immune-related infiltration cells. **(A)** Correlation of immune cells infiltration and risk scores using five different methods. **(B)** Differential infiltration of immune cells between high- and low-risk-score patients. **(C)** Correlation between risk scores and NKT cells infiltration.

In this part, we used the ESTIMATE algorithm to assess the TME immune cell infiltration in patients with BCa. As shown in **Figure 6A**, patients with a high risk score had a lower TME score (stromal score, immune score, and ESTIMATE score) than those in the low-risk group, which illustrated a lower proportion of immune and stromal cell infiltration and higher tumor purity. Then we evaluated the correlation between risk score and the expression of immune checkpoints (ICBs), indicating that most immune checkpoints were significantly low expressed in the high-risk score group (**Figure 6B**) and were negatively correlated with the risk score (**Figure 6C**). The results of immune-related function also revealed that the high-risk group was negatively associated with expression of the chemokine receptor (CCR) and HLA, secreting inflammation-promoting factors and response to IFN-γ (**Figure 6D**). Then, we used five methods to assess the composition of immune infiltration cells. Specifically, natural killer T-cell (NK T cell) infiltration was negatively correlated with the risk score under xCell analysis (**Figures 7A, C**) and was significantly reduced in the high-risk score group (**Figure 7B**).

### Gene Set Enrichment Analysis and Gene Set Variation Analysis

**Figure 8:**
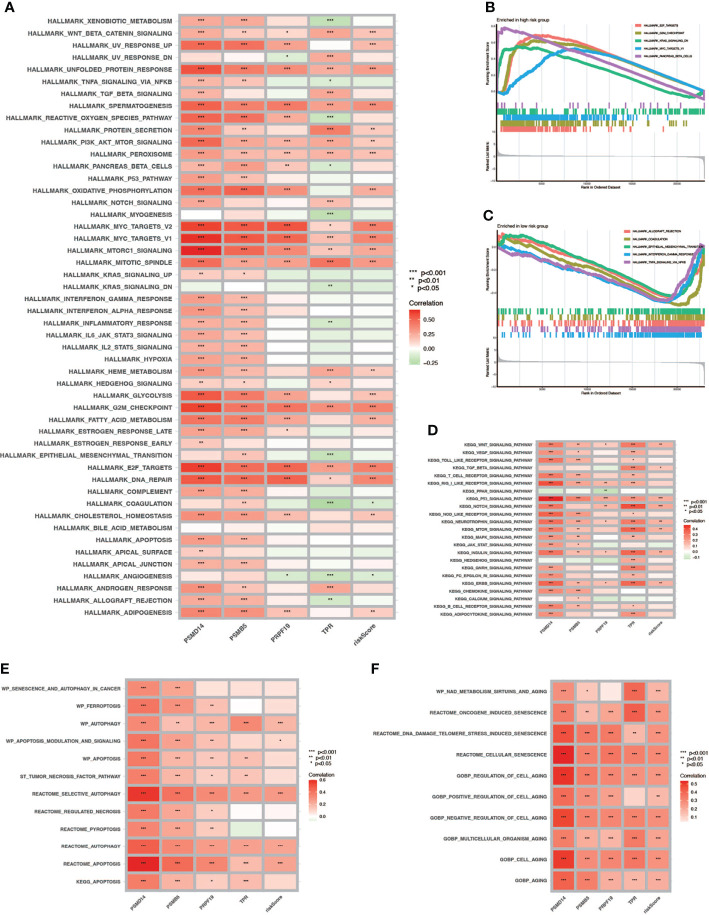
GSVA and GSEA. **(A)** GSVA in Hallmark gene sets. **(B, C)** GSEA in high- and low-risk-score patients. **(D)** GSVA in the KEGG database. **(E)** Correlation between cell death mode and risk score. **(F)** Correlation between cell aging related pathways and risk score (* P < 0.05; ** P < 0.01; *** P < 0.001).

To verify the relationship between risk score and hallmark pathways and cell death mode, we performed GSEA and GSVA using HALLMARK gene sets, Kyoto Encyclopedia of Genes and Genomes (KEGG)_signaling pathways, programmed cell death related gene sets, and cell senescence-related gene sets. As shown in **Figure 8A**, the risk score was positively associated with totally 20 hallmark pathways including PI3K/AKT/mTOR signaling pathway, G2M checkpoint, and fatty-acid metabolism. Then we performed GSVA in different risk score groups (**Figures 8B, C**). Besides, we also performed KEGG analysis and discovered that the risk score was positively correlated with P53, NOTCH, and mTOR signaling pathway (**Figure 8D**). Also, we verified that the risk score was related to some pathways in cell death and cell aging. As shown in **Figures 8E, F**, the risk score was positively correlated with autophagy, apoptosis, and all cell aging-related pathways.

### The Interaction Between Risk Score and Response to Chemotherapy and Immunotherapy

**Figure 9:**
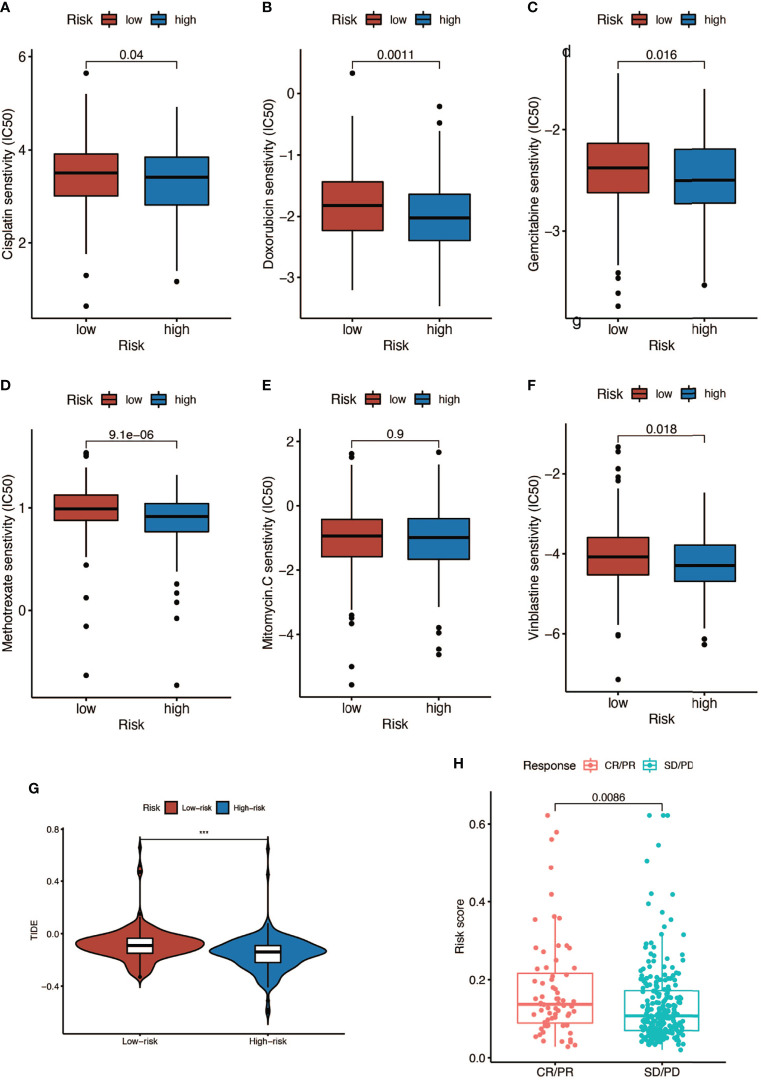
Response to chemotherapy and immunotherapy of patients in the high- and low-risk-score groups. **(A–F)** Comparison of chemotherapy drugs sensitivity in high- and low-risk-score patients including cisplatin, doxorubicin, gemcitabine, mitomycin C, and vinblastine. **(G)** TIDE scores of high- and low-risk-score patients (*** P < 0.001). **(H)** Risk scores of CR (complete response)/PR (partial response) patients and SD (stable disease)/PD (progressive disease) patients after PD-L1 immunotherapy.

We analyzed the response to commonly used chemotherapy drugs in different risk score groups in order to guide clinical medication. As shown in **Figures 9A–F**, the smaller the IC50, the more sensitive patients were to drugs, so patients in the high-risk score were more sensitive to chemotherapy drugs, including cisplatin, doxorubicin, gemcitabine, mitomycin C, and vinblastine. Besides, we also analyzed many other alternative drugs when the above chemotherapy drugs did not work (**Supplementary Figure 3**). Anti-PD-L1 immunotherapy has also been proven effective for patients with metastatic urothelial carcinoma in a multicenter, single-arm phase 2 trial using atezolizumab (IMvigor 210, NCT02108652) (33). Therefore, we applied the IMvigor 210 cohort to verify the response to immunotherapy. The tumor immune dysfunction and ejection (TIDE) score was used to evaluate tumor immunotherapy response, where a smaller TIDE score meant better response to immunotherapy. In **Figure 9G**, patients with a low risk score had a larger TIDE score, indicating less sensitivity to immunotherapy, and in **Figure 9H**, patients in the CR/PR (complete response/partial response) group got a higher risk score than those in the SD/PD (stable disease/progressive disease) group. Overall, patients with a high risk score were more sensitive to immunotherapy.

### Immunohistochemical Analyses of PSMD14, PRPF19, PSMB5, and TPR

**Figure 10:**
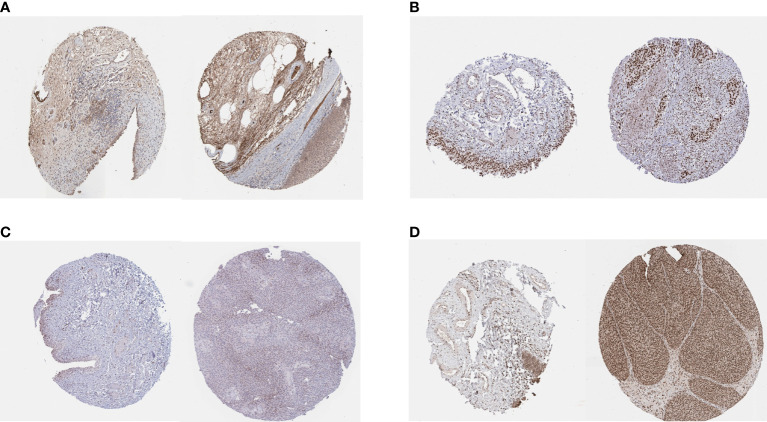
IHC staining of **(A)** PSMD14, **(B)** PRPF19, **(C)** PSMB5, and **(D)** TPR in normal tissues (left) and bladder cancer tissues (right).

We obtained the results of immunohistochemical staining of PSMD14, PRPF19, PSMB5, and TPR in normal tissues and bladder cancer tissues (**Figures 10A–D**) and demonstrated that these four genes were highly expressed in tumor tissues and lowly expressed in normal tissues.

## Discussion

Senescence is a stable state characterized by permanent withdrawal from the cell cycle under endogenous and exogenous stimuli and specific changes in cell morphology and physiology. As a complementary mechanism to programmed cell death, senescence functions like a safeguard to remove dysfunctional or diseased cells and stabilize the body’s internal environment. Senescence has long been considered as a protective mechanism against tumorigenesis since cancerous cells can be induced by senescence and prevented from proliferation and progression (34). However, there is growing evidence that senescence can also contribute to tumor development and progression in certain circumstances and has been regarded as a new hallmark of cancer (35). Studies thought that senescence could promote tumor phenotypes mainly *via* SASP, a phenotype characterized by enhanced secretion of pro-inflammatory cytokines, growth factors, and matrix metalloproteinases (MMPs) in an autocrine or paracrine fashion. These signaling molecules can influence the tumor and other cells in TME and lead to tumor immunity suppression, angiogenesis, tumor proliferation, invasion, and metastasis (36). Studies also found that some tumor cells were in transitory, reversible senescent states and could escape from senescence and reenter the cell cycle, resulting in recurrence and more aggressive tumors (37). Therefore, it is intriguing to deeply explore the exact role of senescence in specific tumor development and prognosis of cancer patients. Nevertheless, limited studies have investigated the effect of senescence in BCa and previous studies only focused on few senescence genes while ignoring their interactions and comprehensive function. Considering the importance of SASP in shaping TME and the development of tumor, we believed it was also valuable and feasible to explore the interaction between TME immune cell infiltration and senescence.

Hence, in this study, using the genome-wide CRISPR screening data acquired from DepMap, we calculated the dependence score *via* the CERES algorithm and screened genes, which was essential to proliferation and survival in BCa cell lines (18). Genome-wide CRISPR has high gene editing specificity and low off-target effect. Therefore, CRISPR is more effective in screening genes. Then we sifted differently expressed genes (DEGs) between paired BCa and normal tissues from these genes using the gene expression data downloaded from TCGA. Afterward, we took the intersection between CRISPR-verified paired DEGs and genes in the CellAge database and finally identified 12 vital senescence genes in BCa, four of which were selected to construct a cell-senescence-regulator-gene prognostic index after applying LASSO and the multivariate COX regression model. To investigate the relationship between senescence index and the prognosis of patients with BCa, we randomly divided the patients in TCGA cohort into training and testing cohorts and calculated the risk score according to the expression of the four senescence genes. No matter for the training cohort and testing cohort (TCGA) or validation cohort (GSE13507), the prognostic senescence index exhibited robust capacity in predicting survival outcomes of patients with BCa. Combining the risk score with other clinical factors (age, gender, stage, and grade), a nomogram was established with better predictive capacity of OS in 1, 3, and 5 years. We further uncovered the interaction between risk score and TMB and their combined effect in predicting the patients’ prognosis. Moreover, we also evaluated the differential TME immune cell infiltration landscape between the two risk subgroups from different perspectives including immune and stromal scores, the purity of tumor, the abundance of immune cells, the expression of immune checkpoints, and the functions of immune cells. We also performed GSVA and GSEA to investigate the interaction between risk score and hallmark pathways. Finally, the role of risk score in heralding the efficacy of immunotherapy and chemotherapy was also investigated.

The four genes which were finally included in the cell-senescence-regulator-gene prognostic index include PSMD14, PSMB5, TPR, and PRPF19, and all belong to senescence inhibitors. PSMD14 is a kind of deubiquitinating enzymes (DUBs) and was reported to be connected with tumor progression and poor prognosis in many types of cancer including neck squamous cell carcinoma (38), non-small cell lung cancer (39), ovarian cancer (40), and hepatocellular carcinoma (41). Gong considered that a high expression of PSMD14 in tumor cells may lead to aging or failure of CD8+ T cells, which may explain the positive correlation between high expression of CD8+ T cells and PSMD14 and poor prognosis in osteosarcoma (42). PSMB5 is a member of the PSMB family and ubiquitin-proteasome system, which was demonstrated to play important roles in tumor progression and immune cell infiltration, especially in breast cancer. PSMB5 was downregulated in M1-polarized THP-1 macrophages. Knockout of PSMB5 with shRNA could promote THP-1 to differentiate into M1 macrophages. It can also inhibit the proliferation and migration of breast cancer cells (43, 44). TPR is essential to a variety of nuclear functions such as the transport of mRNAs and proteins through nuclear pore, chromatin organization, and mitosis, whose mutations were detected in many kinds of cancers and contributed to cancer development and aging (45, 46). PRPF19 participated in DNA damage response and pre-mRNA processing and was reported to be linked with cancer growth in tongue cancer (47) and hepatocellular carcinoma (48) and could regulate cellular senescence in a p53-dependent manner (49). In hepatocellular carcinoma, TP53 mutation may have a positive effect on PRPF19 expression by reducing the promoter methylation of PRPF19 (48). In this article, for the first time, we reported the roles of these genes in the prognosis of BCa. Consistent with other cancers, PSMB5, TPR, and PRPF19 were independent prognostic risk factors in BCa, while PSMD14 seemed to be a protective factor, which was different from other types of cancers. The result needs further verification and the mechanisms need to be explored in the future.

TMB was regarded as a potential biomarker to predict the response to immunotherapy with ICBs. High TMB could predict high ICB efficacy and better overall survival in BCa (6, 50, 51). We found that the high-risk score group had higher TMB compared to the low-risk score group and patients with high TMB had better overall survival, which was consistent with previous studies. We also found that there existed significant distribution differences of TMB in some mutated genes such as TP53 and RB1, both of which were vital TSGs and had higher mutation frequency in the high-risk score group. Previous studies have demonstrated that overexpression of oncogenes could induce cellular senescence and work as a barrier to block tumor growth in the existence of TSGs. However, the mutation of TSGs could lead to senescence escape and was a critical step in tumor progression (13). Therefore, the poor prognosis in the high-risk score group might partially attribute to the loss or inactivation of TSGs.

TME includes not only cancer cells but also immune and stromal cells and plays a vital role in the development of tumor (52). The immune cells consist of tumor-associated macrophages (TAMs), tumor-associated neutrophils (TANs), tumor-infiltrating lymphocytes (TILs), and myeloid-derived suppressor cells (MDSCs). The stromal cells include endothelial cells, cancer-associated fibroblasts (CAFs), and other cells (53). As mentioned above, SASP was characterized by increased secretion of multiple pro-inflammatory and pro-regenerative factors, which could attract and activate immune cells in the TME to clear the senescent cells and maintain the tissue homeostasis or lead to senescence escape and tumor progression, which depends on the kinds of immune cells and factors in the TME. Stromal cells also have the ability of promoting tumor *via* SASP. For example, senescent CAFs were demonstrated to promote tumors through conveying hallmark capabilities to cancer cells in the TME, and senescent fibroblasts in normal tissues could remodel the tissue microenvironments and contribute to tumor invasion and metastasis *via* SASP (35, 36). In our study, we found that a high risk score was connected with a low proportion of immune cells and high purity of tumor cells in the TME of BCa, which means that senescence could interfere with the enrichment of immune cells in TME and lead to the accumulation of tumor cells. We also noticed that the expressions of some immune checkpoints were negatively connected with the risk score with statistical significance, but for those commonly used for immunotherapy target such as PD-1, CTLA-4, and LAG-3, there existed no statistical difference between the high- and low-risk score subgroups. Moreover, we also found that the functions of immune cells in the high-risk score subgroup were partially aberrant compared to the low-risk score subgroup, such as expression of chemokine receptor (CCR) and HLA, secreting inflammation-promoting factors and response to IFN-γ. CCR was important for TAMs to accumulate in the TME, and HLA, also called MHC, was essential to the activation of adaptive antitumor immunity and recognition of tumor cells by NK cells and CD8+ T cells (53). Moreover, IFN-γ could induce Th0 cells and M0 macrophages to differentiate into Th1 and M1 phenotype and exert anti-tumorigenic functions, and it could also induce senescence (53). Besides, we discovered that the NK T cells, which were crucial to the antitumor immunity (54), were negatively connected with the risk score. As a result, the patients in the high-risk score group had a poor survival outcome compared with those in the low-risk score group.

T cells are important for the establishment and maintenance of immune responses. With the process of immunosenescence, T cells could become either anergic, exhausted, or senescent, which would break the immune homeostasis. Exhausted T cells were characterized with high expression of immune checkpoints and decreased production of effector cytokines, while senescent T cells were featured by short telomeres, low expression of co-stimulatory molecules CD28 and CD27, and increased expression of senescence-associated-ß-galactosidase (SA-ß-Gal). CD8+ T cells were more easily influenced by immunosenescence than CD4+ T cells reflected by a remarkably lower number of circulating naïve CD8+ T cells (55). In this article, we found that the risk score was positively correlated with the number of naïve CD8+ T cells in the TME, which means more remarkable immunosenescence in the low-risk score group. Combining the fact that many immune checkpoints were highly expressed in the low-risk group, we thought most T cells were exhausted, which accounts for the high TIDE score and poor immunotherapy outcome using ICBs in this group. Immunotherapy using ICBs has shown promising outcomes in clinical trials among patients with BCa (33, 56). Despite powerful efficacy in some patients, only approximately one-third of the patients see pronounced clinical response to immunotherapeutic intervention (57). It was also reported that immune-mediated cancer control and senescence induction could be achieved and reinforced by the use of ICBs (58). ICBs could enhance the antitumor effect of T cells and NK cells and increase the ability of antigen presentation of dendritic cells to restore senescence barrier. Here, we have demonstrated that risk score could serve as a latent biomarker to predict the response of immunotherapy and screen out the appropriate patients, which was important for precise medicine. Besides, our study found that the high-risk group was positively correlated with a variety of cell death pathways including autophagy. Autophagy is a cellular degradation pathway, which plays a variety of roles in maintaining cell homeostasis. In addition, autophagy also has an impact on cancer initiation, progression, immune infiltration, and metabolism (59). As early as 2009, Narita et al. proposed a new mechanism of autophagy in cell aging: autophagy is activated during aging, which is the same as the result of our study, and its activation is related to the negative feedback of PI3K—mammalian target of rapamycin (mTOR) pathway (60).

Furthermore, we also explored the relationship between risk score and response to chemotherapy. Low doses of chemotherapy itself could induce senescence in cancer cells, which was called chemotherapy-induced senescence. Platinum-based compounds, such as cisplatin, could induce senescence through extensive DNA damage. Moreover, methotrexate and gemcitabine could both induce genotoxic stress by blocking DNA synthesis, thereby inducing cellular senescence (61). Methotrexate, gemcitabine, vinblastine, doxorubicin, and cisplatin were commonly used drugs for MIBC. We found that IC50 for all these drugs were significantly lower in the high-risk score group, which indicated a better response to chemotherapy in this group.

However, some limitations and shortcomings must be addressed in this study. First, the senescence genes were acquired from the CellAge database; as many new senescence genes have been put forward recently, the genes we used for analysis might not be comprehensive enough and this would bring some bias into our studies. Second, the current omics data only provide the level of mRNA and lack protein expression data, which will bring in some inaccuracies. Third, the number of clinical samples is limited and our study lacks external verification from other clinical data sets apart from the public data. Therefore, we are prepared to collect some clinical samples to further verify our conclusions. Finally, the molecular mechanism has not been characterized and further experiments are needed.

In conclusion, our study provided a comprehensive insight into the interaction between cellular senescence, TMB, TME immune cell infiltration, and response to chemotherapy and immunotherapy and interpreted the complicated regulation mechanisms of senescence in BCa. Better understanding and evaluating senescence could be beneficial in selecting appropriate patients, guiding precise therapy, and improving the prognosis of patients with BCa.

## Data Availability Statement

The datasets presented in this study can be found in online repositories. The names of the repository/repositories and accession number(s) can be found in the article/**Supplementary Material**.

## Author Contributions

J-XS and C-QL analyzed the data, wrote the manuscript, and drew the figures; Z-BZ, S-GW, and Q-DX designed the study; J-ZX, YA, M-YX, X-YZ, NZ, S-YM, and H-DH contributed to the critical revision of the manuscript. J-XS, C-QL, Z-BZ, S-GW, and Q-DX contributed equally to this work. All authors contributed to the article and approved the submitted version.

## Funding

This work was supported by the Natural Science Foundation of China (81772729).

## Conflict of Interest

The authors declare that the research was conducted in the absence of any commercial or financial relationships that could be construed as a potential conflict of interest.

## Publisher’s Note

All claims expressed in this article are solely those of the authors and do not necessarily represent those of their affiliated organizations, or those of the publisher, the editors and the reviewers. Any product that may be evaluated in this article, or claim that may be made by its manufacturer, is not guaranteed or endorsed by the publisher.

## Supplementary Material

Supplementary Figure 1 The interaction of 4 genes.

Supplementary Figure 2 The AUC curves of this model in 1, 3, and 5 years.

Supplementary Figure 3 Response to chemotherapy using other drugs in different risk score group.
