# Supplementary figures and images for: A Four-Cell-Senescence-Regulator-Gene Prognostic Index Verified by Genome-Wide CRISPR Can Depict the Tumor Microenvironment and Guide Clinical Treatment of Bladder Cancer
Source: Front Immunol. 2022 Jul 11;13:908068. doi: 10.3389/fimmu.2022.908068 (PMC9312376; doi:10.3389/fimmu.2022.908068)

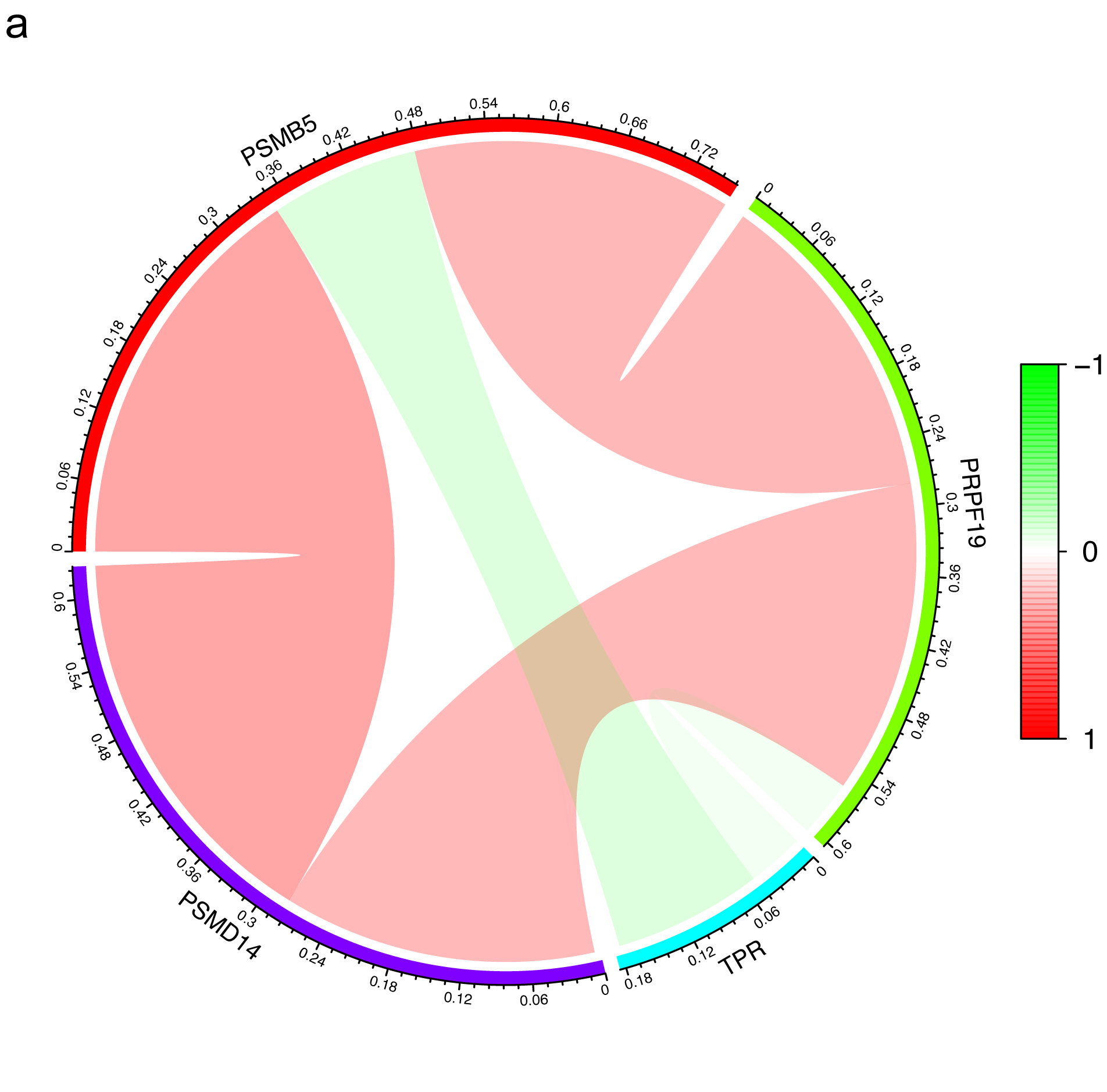

Supplement: Supplementary Figure 1 — The interaction of 4 genes. [file Image_1.tif]

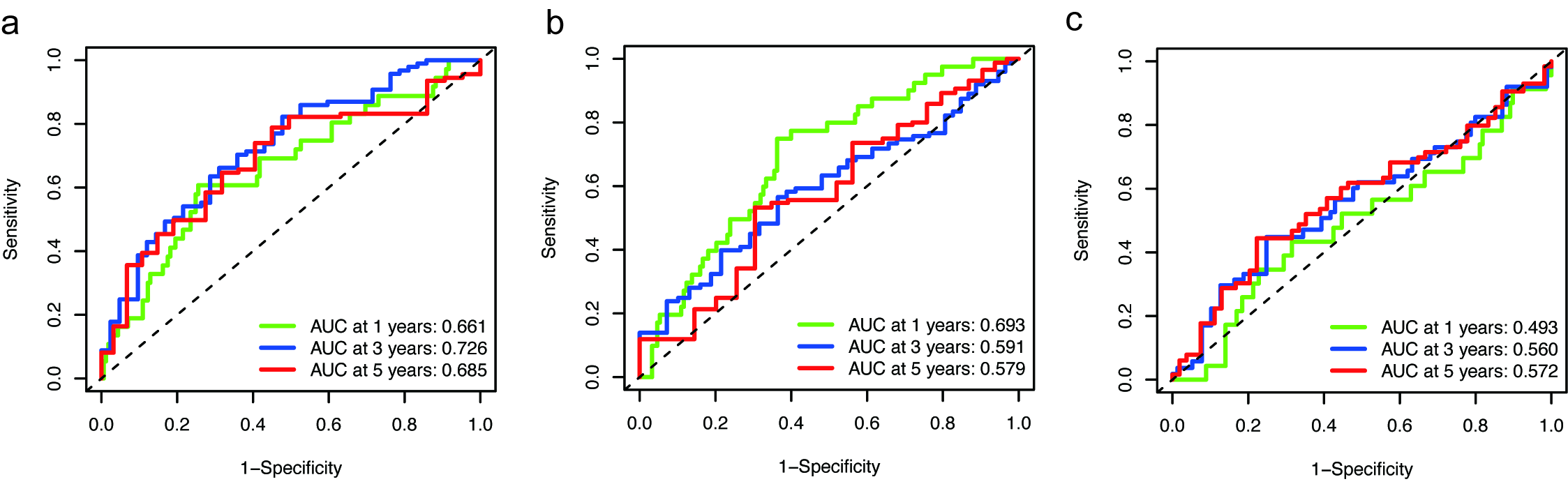

Supplement: Supplementary Figure 2 — The AUC curves of this model in 1, 3, and 5 years. [file Image_2.tif]

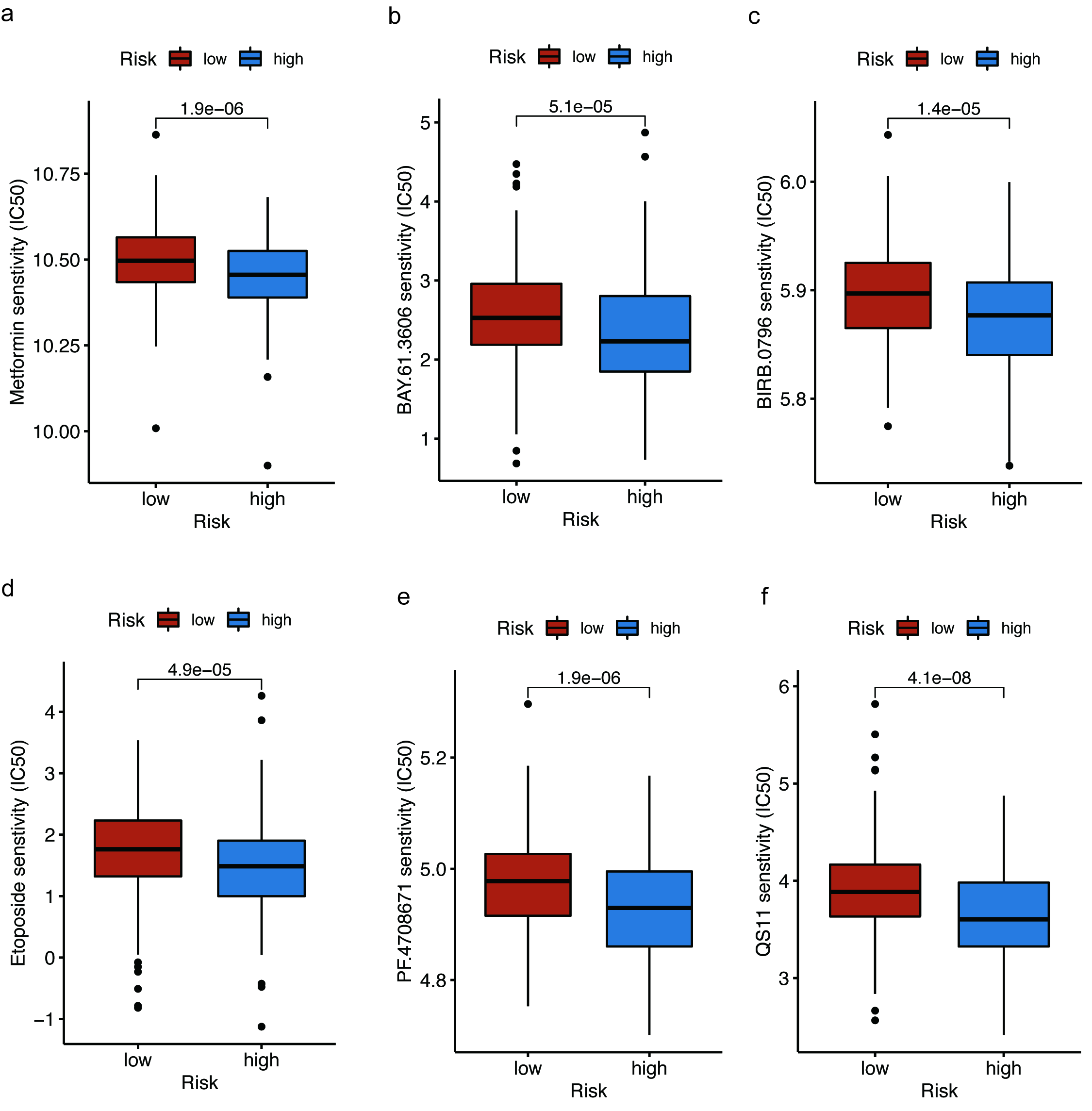

Supplement: Supplementary Figure 3 — Response to chemotherapy using other drugs in different risk score group. [file Image_3.tif]
